# Healthcare expenditure and its socio-demographic and clinical predictors in Australians with poorly controlled asthma

**DOI:** 10.1371/journal.pone.0279748

**Published:** 2023-01-05

**Authors:** Stella T. Lartey, Thomas Lung, Sarah Serhal, Luke Bereznicki, Bonnie Bereznicki, Lynne Emmerton, Sinthia Bosnic-Anticevich, Bandana Saini, Laurent Billot, Ines Krass, Carol Armour, Stephen Jan

**Affiliations:** 1 Norwich Medical School, University of East Anglia, Norwich Research Park, Norwich, United Kingdom; 2 NIHR Applied Research Collaboration, East of England, United Kingdom; 3 The George Institute for Global Health, Sydney, Australia; 4 School of Public Health, Faculty of Medicine and Health, The University of Sydney, Sydney, New South Wales, Australia; 5 Woolcock Institute of Medical Research, Sydney, New South Wales, Australia; 6 School of Pharmacy, The University of Sydney, Sydney, New South Wales, Australia; 7 College of Health and Medicine, University of Tasmania, Hobart, Australia; 8 Tasmanian School of Medicine, Hobart, Tasmania, Australia; 9 Curtin Medical School, Curtin University, Perth, Western Australia, Australia; 10 Faculty of Medicine, University of New South Wales, Sydney, New South Wales, Australia; 11 Central Sydney Area Health Service, Sydney, New South Wales, Australia; Universiti Sains Malaysia, MALAYSIA

## Abstract

**Introduction:**

Asthma has substantial and increasing health and economic burden worldwide. This study aimed to estimate healthcare expenditure and determine the factors that increase expenditure in Australians with poorly controlled asthma.

**Methods:**

Individuals ≥18 years of age with poorly controlled asthma, as determined by a score ≥1.5 on the Asthma Control Questionnaire, were included in the study. Healthcare utilization costs from medical services and medications were estimated over an average follow-up of 12 months from administratively linked data: the Medicare Benefits Schedule and Pharmaceutical Benefits Scheme. A generalized linear model with gamma distribution and log link was used to predict participants’ key baseline characteristics associated with variations in healthcare costs.

**Results:**

A total of 341 participants recruited through community pharmacies were included. The mean (standard deviation, SD) age of participants was 56.6 (SD 17.6) years, and approximately 71% were females. The adjusted average monthly healthcare expenditure per participant was $AU386 (95% CI: 336, 436). On top of the average monthly costs, an incremental expenditure was found for each year increase in age ($AU4; 95% CI: 0.78, 7), being unemployed ($AU201; 95% CI: 91, 311), one unit change in worsening quality of life ($AU35; 95% CI: 9, 61) and being diagnosed with depression and anxiety ($AU171; 95% CI: 36, 306).

**Conclusions:**

In a cohort of Australian patients, characterized by poor asthma control and co-morbidities individuals impose substantial economic burden in terms of Medicare funded medical services and medications. Programs addressing strategies to improve the quality of life and manage co-morbid anxiety and depression and encourage asthma patients’ engagement in clinically tolerable jobs, may result in significant cost savings to the health system.

## Introduction

Asthma is a major cause of disease burden worldwide, with an estimated prevalence of approximately 358 million people and 400,000 deaths in 2015 [1]. This prevalence has increased over the last decade [1–3] and is predicted to reach 400 million cases by 2025 [4]. Asthma is among the top-ranked causes of disability-adjusted life-years (DALYs)–being one of the top 30 conditions in terms of disease burden in under 24 years and over 50 years age groups [5]. This results in substantial healthcare expenditure and overall economic burden [2, 6–8], which has been observed to increase notably with severity and with poorer asthma control [7, 9]. Individuals with poorly controlled asthma tend to have frequent and intense episodes of symptoms, leading to emergency department visits and hospitalization [6, 7, 10].

In Australia, the prevalence of asthma was estimated to be 11.2% across all ages and 11.6% in those over 15 years in 2018. Australia is reported to have one of the highest asthma death rates in the world [11–13]. Whilst the reasons for this high rate are not completely clear it has been posited that environmental factors–particular extremes in weather conditions and impact on air–has played a role [14]. In 2015, the healthcare expenditure associated with asthma in Australia was estimated at approximately $AU1.2 billion [15]. Despite improved treatment and management strategies, the prevalence of poorly controlled asthma remains high [16]. As of 2019, Australia ranked third amongst OECD countries in avoidable hospital admission rates related to asthma [17].

To inform strategies that can efficiently address the needs of patients with asthma, there is a need to identify factors associated with healthcare expenditure in high-risk patients. Particularly expenditure related to medications and non-hospital care, which may be associated with the costs of managing asthma and co-morbidities. This study addresses this issue by using individual-level data collected through the Pharmacy Asthma Service (PAS) trial [18] to estimate healthcare expenditure and its predictors in individuals with poorly controlled asthma. Thus, we investigated whether baseline characteristics collected by the trial, for the cohort, were associated with subsequent healthcare expenditure.

## Methods

The study is a secondary analysis of data from the Pharmacy Asthma Service (PAS) trial. PAS is a pharmacy-based intervention that involves training and support for pharmacists to promote optimal management of patients with asthma. The trial involved a cluster randomized design and was carried out across pharmacies and involved adult patients with poorly controlled asthma presenting to pharmacy. The primary outcome was achievement of asthma control as measured on the Asthma Control Questionnaire (ACQ) at 12 months [19].

The trial was registered with the Australian and New Zealand Clinical Trials Registry (ACTRN12618000313235). The study protocol was approved by the Human Research Ethics Committees of The University of Sydney, Curtin University, and the University of Tasmania. All participants provided written or electronic informed consent. The study was initiated in July 2018 and completed in February 2020.

### Settings

The study was conducted in three states of Australia: New South Wales, Tasmania, and Western Australia. In Australia, the health system is financed by a combination of State/Territory and Federal government sources, private health insurance, and out-of-pocket patient contributions [20, 21]. State, Territory, and local governments deliver and manage public health services. Medical services are subsidized by the Australian Federal government through the Medicare Benefits Schedule (MBS) [22]. The MBS records government and patient out-of-pocket expenditure (government legislated copayments) on general practitioner and specialist visits and diagnostic tests. The Federal government subsidizes prescribed medicines through the Pharmaceutical Benefits Scheme (PBS), with most participants contributing co-payment for each prescription [21, 23].

### Study population

Individuals in the study were identified and recruited by pharmacy staff through community pharmacies and were eligible if they fulfilled the following criteria: were ≥18 years of age with poorly controlled asthma, as determined by a score ≥ 1.5 on the ACQ [24, 25]; were able to communicate with the pharmacist in English; were a regular patron of the pharmacy (receiving medications from that pharmacy for the previous 12 months); and self-managed their medication (as determined by the pharmacist). Participants were not eligible to participate in the program if they had a high dependence on medical care (more than five co-morbidities and specialist care), confirmed diagnosis of chronic obstructive pulmonary disease (as reported by the participant), or terminal illness [18, 26].

### Outcomes

The primary outcome was the total cost of medical and pharmaceutical services derived from MBS and PBS data. Services Australia (formerly the Department of Human Services) is acknowledged for supplying the Pharmaceutical Benefits Scheme (PBS) and Medicare Benefits Schedule (MBS) information. This study used the health system perspective for the costing of medical services and prescribed pharmaceuticals. Because of differences in length of follow-up between participants, an average monthly cost was estimated. Expenditure from baseline to the end of the 12 months of trial period was estimated in this study. Study data were linked to administrative MBS and PBS data of consenting participants during the trial; the average follow-up was 12 months. MBS costs include *all* non-public hospital medical services, specifically, general practitioner visits, private outpatient visits, specialized care, and tests and examinations (e.g., imaging and laboratory services). PBS costs were those of *all* prescription medications dispensed mainly through community pharmacies. The Australian Bureau of Statistics health consumer price index was used to inflate costs incurred in earlier years. All costs are presented in 2020 Australian dollars [27] ($AU1~$US 0.6906 [28]).

#### Explanatory variables

Explanatory variables were specified based on Andersen’s behavioral model of factors that facilitate or impede health resource utilization and those that affect expenditure [29, 30]. In particular, the Andersen model shows that the individual utilizes health resources depending on three sets of characteristics: (1) *predisposing factors*, also known as the socio-cultural characteristics of the person, including age, gender, educational level, occupation, location; (2) *enabling factors* focusing on the logistical requirements needed to seek care, such as individual or family income, having health insurance, availability of health personnel and facilities; and (3) *need factors* that are the immediate cause for which a person seeks care, including disease or disability. We used this model to select the explanatory baseline variables in this study: age, gender, educational level, occupation, location, smoking status, number of self-reported hospital admissions and accidents or emergency visits in the previous 12 months, and self-reported diagnosis with hay fever, depression, and anxiety (not necessarily related with asthma). Additionally, we included clinical factors that characterize asthma, including the age of asthma onset, ACQ score, rhinitis control assessment test (RCAT) score, and the impact of asthma on the quality of life questionnaire (IAQLQ) score as a measure of the quality of life.

Age was defined as a continuous variable, and gender was categorized as male/female. Educational status was grouped into ‘no formal education’, ‘up to high school education’ and ‘above high school education’. Employment status was categorized as ‘full-time’, ‘part-time’, or ‘no formal employment’, and because of the relative populations state of residence was grouped into ‘Tasmania plus Western Australia’ versus ‘New South Wales’. Smoking status, diagnosis with hay fever, depression, and anxiety were all dichotomized. The age of asthma onset was grouped into less than age 35 years, and 35 years and above. Self-reported hospital admissions, emergency presentations, ACQ, RCAT, and the IAQLQ scores were treated as continuous variables. Asthma Control Questionnaire scores lie between 0 (totally controlled) and 6 (extremely poorly controlled). A score of 1.5 or greater is considered an indication of poorly controlled asthma [25]. Rhinitis Control Assessment Test scores lie between 6 and 30, with lower scores indicative of more severe allergic rhinitis. Participants scoring ≤21 are considered clinically “symptom uncontrolled”; those scoring >21 are considered “symptom controlled” [31]. The Impact of Asthma on Quality of Life Questionnaire scores lie between 0 and 10, with higher scores indicative of lower quality of life [32].

### Statistical analysis

We undertook descriptive statistics of both outcome and explanatory variables. Means and standard deviations were reported for continuous variables, while percentages were reported for categorical variables. Associations between key baseline variables and monthly average healthcare expenditure were analyzed using regression techniques. Due to the strong non-normality (right-skewed) in the distribution and presence of outliers in the cost data, multivariable analyses were performed using generalized linear model (GLM) specification from the gamma family and log link [33, 34], with clustering by pharmacy (in which the participants were recruited) taken into account. Detail model diagnostics have been provided in the Methods Appendix in S1 File). Incremental expenditure was estimated: for categorical variables, this was the difference in the adjusted expenditure between the category of interest versus the reference category; for continuous variables, this was the change in average expenditure per unit increase in that variable [34, 35].

Interaction terms were generated between variables that proved to be significant in the main model used for the multivariable analyses. Data from statistically significant interactions are presented in the results section. It is worth noting that margins command used to retrieve incremental expenditure after using GLM with log link is applicable for interaction between either two factor variables, two continuous variables or for a specified value (point) for the continuous variable when the interactions are between a factor variable and a continuous variable. Thus, incremental expenditures were shown for the interaction between two factor variables or two continuous variables or at a specified value when the interaction was between a factor variable and a continuous variable.

Out of 381 participants in the trial, 40 individuals were excluded from the study due to invalid consent to the linkage (n = 3), lack of consent to linkage to the administrative data (n = 32), missing MBS and PBS data for the period (12 months) and other explanatory variables (n = 5). The final sample was 341. The trial, based on a cluster randomized design, was powered (90%) to detect a 20% absolute improvement in the primary outcome—proportion of patients with controlled asthma at 12 months. The estimated sample size was 80 pharmacies (40 per arm), each with seven patients (targeted sample size = 560 patients). Other assumptions were no more than 30% with controlled asthma in the comparator group, an intra-cluster correlation of 0.1, 20% patient withdrawal and 15% pharmacy withdrawal [19].

Due to extremely high costs incurred by a number of individuals, a positive skewness in the expenditure data was observed. Therefore, a sensitivity analysis was conducted, in which the individuals with the 5% highest monthly costs were excluded from the data and the data re-analyzed. A two-tailed p-value<0.05 was determined as statistically significant. All analyses were performed using STATA v.16 (Stata Corp., Lakeway Drive, College Station, TX, USA).

## Results

**Fig 1:**
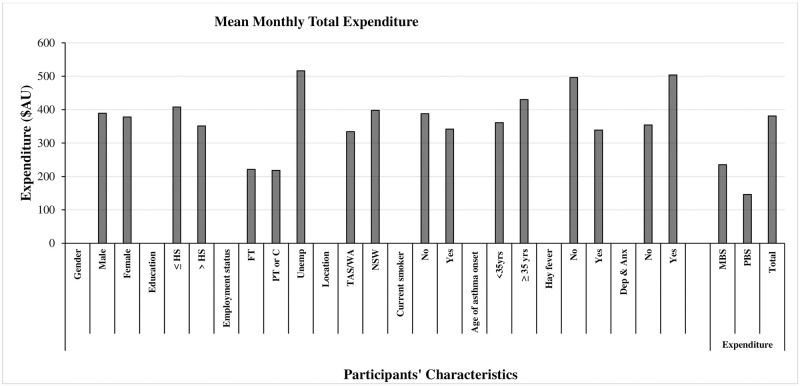
Mean monthly health care expenditure summary by characteristics and by MBS and PBS categories of the study participants ($AU). This presents the unadjusted monthly mean total expenditure of participants by characteristics. HS denotes high school; FT-full time; PT-part time; C-casual; Unemp-unemployed; TAS-Tasmania; WA-Western Australia; yrs-years; Dep & Anx-depression and anxiety; MBS- Medicare Benefits Schedule; PBS-Pharmaceutical Benefits Scheme; and the total is the summation of MBS and PBS expenditure.

The mean (standard deviation, SD) age of participants was 56.6 years (17.6), approximately 71% were females, and 48% had above-high-school education (Table 1). Of the total, 71% reported their age of asthma onset as less than 35 years. The mean ACQ score at baseline was 2.5 (0.9), the mean baseline RCAT score was 15.3 (10.0), and the mean baseline IAQLQ score was 3.4 (2.0). Fig 1 summarizes the unadjusted expenditure per participant defined by their characteristics. Healthcare expenditure was higher among the unemployed (mean: $AU516; SD: $AU468), those without hay fever (mean: $AU496; SD: $AU553), and participants with the age of asthma onset at 35 years or above (mean: $AU430; SD: $AU376).

**Table 1:** Baseline demographic and clinical characteristics of study participants.

Characteristic	All (%)
Number of participants, n	341 (100)
Age, years (Mean, SD)	56.6 (17.6)
Gender	
Male	100 (29.3)
Female	241 (70.7)
Education	
Up to high school	179 (52.5)
Above high school	162 (47.5)
Employment status	
Full-time	79 (23.2)
Part-time or casually employed	76 (22.3)
Unemployed	186 (54.6)
Location	
TAS/WA	92 (27.0)
NSW	249 (73.0)
Current smoker	
No	292 (85.6)
Yes	49 (14.4)
Age of asthma onset	
<35 years	241 (70.7)
35 years	100 (29.3)
Hospital admissions in past 12 months	0.28 (1.1)
Emergency department (ED) presentations in past 12 months	0.53 (2.0)
Asthma control score (1.5)	2.50 (0.9)
Rhinitis Control Assessment Test	15.3 (10.0)
IAQLQ score	3.4 (2.0)
Hay fever	
No	91 (26.7)
Yes	250 (73.3)
Depression and anxiety	
No	280 (82.1)
Yes	61 (17.9)

Data are mean (standard deviation) for continuous variables and percentages (sample, n) for categorical variables.

Results from the univariable and multivariable analyses of the total monthly expenditure are presented in Table 2. The univariable analyses showed a year increase in age ($AU7; 95% CI: 4, 10; *p*<0.001), being unemployed ($AU295; 95% CI: 186, 403; *p*<0.001) compared to having a full-time job, one unit increase in ACS ($AU80; 95% CI: 25, 134; *p* = 0.004) or IAQLQ score ($AU41; 95% CI: 14, 67; *p* = 0.003) and being diagnosed with depression and anxiety ($AU150; 95% CI: 6, 293; *p* = 0.041) was associated with a significant incremental expenditure on top of the mean monthly expenditure.

**Table 2:** Predicted increase in monthly expenditure associated with a range of baseline characteristics in participants with poorly controlled asthma ($AU).

Characteristic	Univariable		Multivariable	
	Monthly Incremental Expenditure (95% CI)	*p-*value	Monthly Incremental Expenditure (95% CI)	*p-value
Age, years	7.1 (3.8, 10.4)	<0.001	4.0 (0.8, 7.1)	0.015
Gender (Ref: Male)				
Female	-10.6 (-132.9, 111.8)	0.866	8.4 (-96.1, 112.9)	0.875
Education (Ref: Up to high school)				
Above high school	-56.7 (-137.1, 23.8)	0.168	40.9 (-32.4, 114.1)	0.275
Employment status (Ref: Full-Time Employed)				
Part Time or casually employed	-3.4 (-105.5, 98.7)	0.947	-45.1 (-143.9, 53.8)	0.372
Unemployed	294.8 (186.3, 403.2)	<0.001	200.7 (90.7, 310.6)	<0.001
Location (Ref: WA/TAS)				
NSW	64.2 (-52.7, 181.1)	0.282	32.5 (-58.3, 123.3)	0.483
Current smoker (Ref: No)				
Yes	-46.0 (-150.4, 58.4)	0.388	-31.0 (-163.8, 101.9)	0.648
Age of asthma onset (Ref: <35 years)				
≥35 years	69.7 (-37.1, 176.4)	0.201	-8.8 (-118.9, 101.3)	0.875
Hospital admissions past 12 months	20.2 (-1.7, 42.0)	0.071	28.6 (-14.8, 72.0)	0.196
ED presentations past 12 months	6.0 (-0.9, 13.0)	0.090	-11.8 (-33.1, 9.7)	0.283
Asthma Control Score	79.6 (25.1, 134.1)	0.004	29.4 (-26.5, 85.2)	0.303
Rhinitis Control Assessment Test	-4.7 (-10.9, 1.5)	0.138	7.5 (-4.2, 19.2)	0.210
IAQLQ score	40.5 (13.9, 67.1)	0.003	35.0 (9.0, 61.0)	0.008
Hay fever (Ref: No)				
Yes	-157.4 (-311.8, -3.1)	0.046	-263.9 (-562.8, 35.0)	0.083
Depression and anxiety (Ref: No)				
Yes	149.5 (6.0, 293.1)	0.041	171.3 (36.2, 306.4)	0.013
Intercept	-	-	386.3 (336.1, 436.5)	<0.001

*p-value<0.05 was determined as statistically significant.

Variables included in the multivariable model included age, gender, education, employment, and smoking statuses, location of residence, age of asthma onset, hospital admission, emergency department visits, ACS, RCAT, and IAQLQ scores, having hay fever, and depression, and anxiety or otherwise. In the multivariable analysis, the adjusted mean monthly expenditure was $AU386 (95% CI: 336, 436; *p*<0.001). A statistically significant adjusted incremental expenditure on top of the mean monthly expenditure per participant was associated with a year increase in age ($AU4; 95% CI: 0.78, 7), being unemployed ($AU201; 95% CI: 91, 311) compared to having a full-time job, an increase IAQLQ score ($35; 95% CI: 9, 61), and being diagnosed with depression and anxiety ($AU171; 95% CI: 36, 306). Conversely, the results show that being female compared to male, having a part-time job compared to having a full-time job, location, smoking status, age of asthma onset, hospital, or AE visits, asthma control or RCAT scores, and having hay fever were not significantly associated with variation in expenditure.

### Results from interactions

Results from the interaction terms are presented in Table 3. Interactions were generated between age and employment status, age and IAQLQ scores, employment status and IAQLQ scores, and employment status and depression and anxiety variables. For the age and employment status interaction, age was treated as a continuous variable. The minimum age was 19 years and maximum age was 101 years. On the basis that asthma has high disease burden in under 24 years and over 50 years age group [5], the average age for observations 1) <24 years, i.e., 21 years; 2) 24 year≥Age≤50 years, i.e., 38 years; and 3) >50 years, i.e., 67 years in the data were specified for the incremental expenditure. *Ceteris paribus*, all the categories in the interaction term were significantly associated with high incremental expenditure and higher age had higher expenditure. However, being unemployed in each age group (at age 21 years: $AU273; 95% CI: 142, 403; at age 38 years: $AU 347; 95% CI: 238, 456; and At 67 years: $AU524; 95% CI: 441, 608) demonstrated significant high incremental expenditure with substantially high expenditure observed at age 67 (i.e., in those above 50 years old). Age and IAQLQ scores interaction showed no significant associations.

**Table 3:** Results from interaction terms.

Characteristic	Multivariable	
	Monthly Incremental	*P-value
	Expenditure (95% CI)	
Age (years)* Employment status		
At 21 years and Full -time	188.1 (64.6, 311.6)	0.003
At 21 years and Part -time	182.8 (103.4, 262.2)	<0.001
At 21 years and Unemployed	272.8 (142.4, 403.1)	<0.001
At 38 years and Full -time	219.1 (125.9, 312.2)	<0.001
At 38 years and Part -time	201.4 (144.3, 258.4)	<0.001
At 38 years and Unemployed	347.2 (237.5, 455.8)	<0.001
At 67 years and Full -time	284.1 (153.0, 415.2)	<0.001
At 67 years and Part -time	237.6 (159.3, 315.9)	<0.001
At 67 years and Unemployed	524.0 (440.5, 607.5)	<0.001
Age (years)* IAQLQ score	-0.81 (-2.09, 0.47)	0.214
IAQLQ score* Employment status		
IAQLQ score = 1.1 and Full -time	124.5 (85.6, 163.4)	<0.001
IAQLQ score = 1.1 and Part -time	225.7 (133.8, 317.7)	<0.001
IAQLQ score = 1.1 and Unemployed	374.0 (289.6, 458.5)	<0.001
IAQLQ score = 5.7 and Full -time	662.3 (127.6, 1197.0)	0.015
IAQLQ score = 5.7 and Part -time	227.3 (148.3, 306.3)	<0.001
IAQLQ score = 5.7 and Unemployed	547.4 (432.3, 662.6)	<0.001
IAQLQ score = 8.4 and Full -time	1766.6 (-648.4, 4181.6)	0.152
IAQLQ score = 8.4 and Part -time	228.2 (82.6, 373.8)	0.002
IAQLQ score = 8.4 and Unemployed	684.6 (440.8, 928.4)	<0.001
Depression and anxiety (D&A)* Employment status		
No D&A and Full -time	255.4 (162.1, 348.6)	<0.001
No D&A and Part -time	179.2 (143.0, 215.3)	<0.001
No D&A and Unemployed	422.2 (347.7, 496.8)	<0.001
Yes D&A and Full -time	182.0 (105.2, 258.8)	<0.001
Yes D&A and Part -time	520.3 (158.4, 882.1)	0.005
Yes D&A and Unemployed	695.3 (473.1, 917.5)	<0.001

Regarding the interaction between employment status and IAQLQ scores (minimum = 0.25, maximum = 9.875), three averages of IAQLQ in the data were specified for the incremental expenditure on the assumption that 1) <5 units score was poor (average: 1.1); 2) 5≥IAQLQ≤7 was moderate (average: 5.7); and >7units scores as good (average: 8.4). The results showed that almost all the categories in the interaction term were significantly associated with high incremental expenditure. Noting that except for having a moderate IAQLQ score and in full-time employment (IAQLQ score = 5.7: $AU 662; 95% CI: (128, 1197), being unemployed with poor (IAQLQ score = 1.1: $AU 374; 95% CI: 290, 459) or good (IAQLQ score = 8.4: $AU 685; 95% CI: 441, 928) IAQLQ score was associated with significantly higher incremental expenditure.

The interaction between employment status and depression and anxiety also showed that all the categories in the interaction term were significantly associated with high incremental expenditure. However, being unemployed with ($AU695; 95% CI: 473, 918) or without ($AU422; 95% CI: 348, 497) depression and anxiety was associated with highly substantial incremental expenditure. Results from the interaction terms showed that while increasing age, worsening IAQLQ scores and being diagnosed with depression and anxiety increased healthcare expenditure, being unemployed under any circumstance in this study was associated with highly substantial incremental expenditure.

### Sensitivity analysis

A sensitivity analysis in which the top 5% of individuals with the highest expenditure were excluded is presented in Tables 4 and 5. Findings in the multivariable analysis show a lower average expenditure compared to the estimate in the full sample in Table 5. One year increase in age ($AU4; 95% CI: 2, 6) remained associated with an incremental expenditure of $AU4 but became statistically significant. The incremental expenditure associated with being unemployed ($AU163; 95% CI: 97, 230) and a unit increase in IAQLQ score ($AU23; 95% CI: 8, 38) were lower but remained statistically significant. Having depression and anxiety was no longer associated with increased expenditure, which perhaps suggest its effect was strongest in patients with very high levels of expenditure (and thus severity of illness).

**Table 4:** Monthly Average Total Expenditure summary by characteristics for the study participants.

Characteristic	Average Monthly Expenditure, ($AU)	
	Average (Standard deviation)	Median (IQR)
Gender		
Male	314 (278)	222 (109, 444)
Female	300 (261)	221 (109, 405)
Education		
Up to high school	340 (279)	276 (127, 499)
Above high school	263 (245)	177 (91, 358)
Employment status		
Full-time	161 (175)	112 (51, 224)
Part-time or casually employed	200 (185)	136 (75, 262)
Unemployed	413 (282)	350 (192, 581)
Location, n		
TAS/WA	302 (286)	198 (96, 396)
NSW	304 (258)	227 (114, 410)
Current smoker		
No	304 (269)	218 (110, 407)
Yes	305 (247)	249 (103, 422)
Age of asthma onset		
<35 years	271 (248)	197 (92, 379)
≥ 35 years	381.95 (291)	317 (146., 581)
Hay fever		
No	342.5 (252.8)	289 (133, 493)
Yes	290.71 (269)	201.7 (96, 387)
Depression and anxiety		
No	291 (252)	216 (109, 389)
Yes	366 (318)	309 (114, 567)
MBS-Schedule	200 (182)	147 (72, 259)
PBS-Benefits	104 (141)	55 (11, 143)
MBS+PBS	304 (266)	221 (109, 409)

Sensitivity analysis was done by excluding the top 5% highest total average monthly costs from the sample.

**Table 5:** Predictors of monthly average total expenditure in participants diagnosed with asthma. Predicted incremental costs ($AU) are presented.

Characteristic	Multivariable	
	Monthly Incremental Expenditure (95% CI)	*P-value
Age, years	3.9 (1.8, 6.0)	<0.001
Gender (Ref: Male)		
Female	-25.5 (-89.3, 38.3)	0.434
(Ref: Up to high school)		
Above high school	-15.3 (-66.3, 35.7)	0.556
Employment status (Ref: Full-Time Employed)		
Part-Time or casually employed	10.8 (-53.4, 75.0)	0.372
Unemployed	163.3 (97.1, 229.5)	<0.001
Location (Ref: WA/TAS)		
NSW	-21.5 (-88.9, 46.0)	0.533
Current smoker (Ref: No)		
Yes	-6.9 (-87.3, 73.4)	0.866
Age of asthma onset (Ref: <35 years)		
≥35 years	10.9 (-60.9, 82.6)	0.766
Hospital admissions past 12 months	32.4 (-9.5, 74.3)	0.130
ED presentations past 12 months	-11.2 (-28.8, 6.4)	0.212
Asthma control score	11.6 (-19.8, 42.9)	0.47
Rhinitis Control Assessment Test	-0.01 (-8.0, 8.0)	0.997
IAQLQ score	23.0 (7.8, 38.2)	0.003
Hay fever (Ref: No)		
Yes	-10.6 (-186.0, 164.8)	0.906
Depression and anxiety (Ref: No)		
Yes	93.2 (-5.5, 191.9)	0.064
Intercept	307.2 (282.6, 331.7)	<0.001

*P value<0.05 was determined as statistically significant.

Sensitivity analysis was done by excluding the top 5% highest monthly average total expenditure from the sample.

Overall, the results on interactions showed similar trends as those in the main regression model but with slightly lower incremental expenditure (Table 6: All data not presented). However, while in the sensitivity analysis, depression and anxiety was not significantly associated with expenditure, the interaction between employment status and depression and anxiety showed significant results. It also showed that all the categories in the interaction term were significantly associated with high incremental expenditure. However, being unemployed with ($AU470; 95% CI: 357, 583) or without ($AU334; 95% CI: 295, 374) depression and anxiety was associated with substantial incremental expenditure.

**Table 6:** Results from interaction terms.

Characteristic	Multivariable	
	Monthly Incremental	*P-value
	Expenditure (95% CI)	
Employment status (Ref: Full -time)		
Part Time or casually employed	9.3 (-44.53439 63.05)	0.736
Unemployed	169.9 (109.7, 230.2)	<0.001
Depression and anxiety (Ref: No)		
Yes	100.5 (8.5, 192.5)	0.032
Depression and anxiety (D&A)* Employment status		
No D&A and Full -time	185.6 (138.5, 232.6)	<0.001
No D&A and Part -time	183.2 (145.7, 220.7)	<0.001
No D&A and Unemployed	334.1 (294.5, 373.7)	<0.001
Yes D&A and Full -time	194.9 (105.8, 284.0)	<0.001
Yes D&A and Part -time	260.9 (151.5, 370.3)	<0.001
Yes D&A and Unemployed	469.5 (356.5, 582.6)	<0.001

Results from other interaction terms are not presented.

## Discussion

This study estimated health care expenditure and identified predictors of incremental expenditure in a cohort of Australian adults with uncontrolled asthma. We calculated an average monthly asthma-related expenditure of $AU386 per participant. In terms of how baseline characteristics influenced healthcare expenditure, the multivariable analysis showed that expenditure in this adult population was positively associated with age, unemployment, lower quality of life score, and depression and anxiety, highlighting these as indicators of heightened healthcare needs.

Our analysis underscores the substantial economic burden of poorly controlled asthma on the Australian health system. As previous studies have focused on different aspects of healthcare expenditure components, used different methods, and have been undertaken in different healthcare systems, caution is needed in comparing our results with previous estimates. Nonetheless, our findings corroborate existing evidence that asthma is associated with higher healthcare expenditure, with the highest burden found in participants with poorly controlled or severe asthma [7, 35–39]. While our findings show that those with poorer asthma control have higher expenditure (as reflected in the univariable analysis), the association between expenditure and asthma control was no longer significant in the multivariable analysis. This suggests that the association between asthma control and expenditure may have been mediated by other significant factors in the multivariable model, such as age, unemployment, quality of life, and depression and anxiety. Among all significant healthcare expenditure predictors in this study, our interaction terms showed that unemployment was a major factor associated with the highest expenditure. Furthermore, because individuals in the study had reported poor asthma control (a score ≥1.5 on the Asthma Control Questionnaire) at study entry there may have been a threshold effect of asthma control on expenditure.

The literature shows that annual average expenditure varies immensely, ranging from $US200 to $US7,000 (~$AU290-$AU10,136) in participants with controlled asthma and approximately $US2,000 to $US12,000 (~$AU2,896-$AU17,376) in those with poorly controlled or severe asthma [7, 35–39]. Our study has shown that the average monthly expenditure of $AU386, when extrapolated to an annual value of $AU4,632 per person per year, is consistent with findings from other studies conducted in OECD countries [7, 35–39]. The expenditure and, by extension, the cost burden estimated in this study, suggests there is potential for effective asthma management strategies and strategies to address co-morbidities, to achieve substantial economic savings for the health system. Previous studies have shown that clinical factors formed the majority of cost drivers [35, 37]. They included more severe or poorly controlled asthma, general physician visits, prescription medication, hospitalizations, hospital-based outpatient visits, emergency department visits, and the presence of co-morbidities [35, 37]. While predisposing factors such as urban residence setting and being current smokers were associated with higher costs, costs associated with age have varied [3, 7, 35, 36, 40]. Jacob *et al*. found in a German cohort that a participant diagnosed with intermittent or persistent asthma incurred a higher direct cost as age increased and were the highest in those over 18 years [36]. In Japan, age and co-morbidities exacerbated severe asthma, leading to increased resource use and costs [7]. In our study, age significantly predicted higher average monthly costs of $AU390 ($AU4 monthly, plus an average monthly expenditure of $AU386), and thus extrapolated, an annual value of $AU4,680 is expected to be incurred with each year in age. A body of literature has shown that increasing age is associated with increasing risk for frailty, disability, and therefore, healthcare costs, be it direct or indirect [41]. Thus, our finding of increasing expenditure with age reinforces existing literature highlighting the critical need to address financial challenges to the health care system that the pressures of an aging population and increasing asthma prevalence in Australia pose [42, 43].

The data also showed that an increase by one unit in IAQLQ score in poorly controlled asthma patients was significantly associated with a $AUD35 (~$AUD420 increase monthly, and extrapolated annual value of $AU5,052 for these patients) increase in healthcare expenditure; indicating that lower quality of life among uncontrolled asthma patients is associated with higher costs. This association between low quality of life and increased costs in individuals with poor asthma control corroborates previous studies and is linked to frequent and life-threatening attacks, increased co-morbidities, and high medical treatment requirements [3, 10]. Conversely, improving asthma control has also been associated with improved quality of life and, subsequently, work productivity [2, 7, 35]. The data from our study reinforce this relationship between lower quality of life and higher costs amongst individuals with poorly controlled asthma. It suggests more broadly that poor asthma control tends to occur in individuals alongside other significant health challenges–one such challenge being highlighted in another aspect of our findings, the positive association between costs and co-morbidity in the form of depression and anxiety. Even though association with co-morbidity was no longer significant when the top 5% of high-cost individuals were dropped in the sensitivity analysis, the interaction between employment status and depression and anxiety showed significantly positive association with expenditure. Thus, in general, our finding concurs with broader evidence indicating that co-morbidities predict higher costs in asthma patients [35, 44], with perhaps a stronger association the greater the severity of illness.

That costs were found to be positively associated with unemployment in our study supports existing literature [37–39]. All our interaction terms showed that being unemployed under any circumstance (based on variables shown to have significant associations in this study) was associated with highly substantial incremental expenditure. Previous studies have shown that patients with poorly controlled asthma, compared to persons without asthma, were 4.6 times more likely to be hospitalized, twice as likely to visit emergency departments, have higher absenteeism and activity limitation, and be unemployed [3, 45, 46]. We found the positive correlation between unemployment and health care costs also holds when costs are defined as non-hospital health care and prescribed medicines. This suggests that access to non-hospital health care in Australia if used may enable higher expenditure within this group in the short term but may be cost-saving in the long-term since higher costs from hospital admissions which are associated with higher costs would be avoided. Whether this would be the case in Australia or would indicate potential excess needs in this population are questions to be addressed in future studies.

While ACQ was not significantly associated with healthcare expenditure, it should be noted that in a cohort of individuals (i.e., in this study) characterized by poor asthma control (score ≥ 1.5 on the ACQ), there were overall high levels of expenditure recorded. The non-significant finding indicates that within this cohort who exceeded the threshold of ≥ 1.5 on the ACQ, variation in ACQ score was not associated with expenditure. Further studies using a comparator with low ACQ thresholds in this population may give a fuller picture of the excess financial burden associated with this patient population.

There are some limitations in this study. The analysis of healthcare costs includes only those recorded to the MBS and PBS and excludes hospitalization costs, privately funded services, over-the-counter medicines, alternative therapies, and indirect costs (e.g., lost productivity to access healthcare, travel costs, and productivity costs due to absenteeism). On the other hand, our analysis captures all expenditure in MBS and PBS, not only those related to asthma, thus enabling analysis of the burden associated with co-morbidity alongside asthma. Seventy-one percent of our sample of adults with poor asthma control were women. While the evidence generally indicates that adult females generally have a higher prevalence of asthma and severe asthma than adult males [47], as well as asthma mortality, there is no evidence that this sex disparity in poor asthma control is reflected in the general population [48]. The strength of this study is that it is based on administratively linked data, thereby providing unbiased estimates of costs for individuals for the study period. Another limitation is that in the trial, it was observed that between baseline and 12 months, individuals in both arms achieved significant improvements in asthma control from baseline. To the extent that this may be due to participation in the study, rather than the natural course of the condition, the costs reported here would be an under-estimate.

## Conclusion

This study has confirmed the substantial direct healthcare costs associated with poorly controlled asthma in the Australian population particularly among unemployed asthma patients. This research suggests the need for effective strategies to improve the quality of life, manage co-morbidities and encourage asthma patient to engage in clinically tolerable jobs, as these will potentially yield substantial cost savings to the health sector.

## Supporting information

S1 File (DOCX)
