# Supplementary material for: Healthcare expenditure and its socio-demographic and clinical predictors in Australians with poorly controlled asthma
Source: PLoS One. 2023 Jan 5;18(1):e0279748. doi: 10.1371/journal.pone.0279748 (PMC9815839; doi:10.1371/journal.pone.0279748)
Supplement: S1 File — (DOCX) [file pone.0279748.s001.docx]

Methods Appendix

We checked and conducted detailed distribution analysis of the average monthly expenditure data before resorting to use the multivariable GLM recommended for skewed cost data ([1](#_ENREF_1)). First, we examined the distribution of our average monthly expenditure data, and it did not meet assumptions of normality requirement for simple linear regression as demonstrated by the symmetry plot (Methods Appendix Fig 1) below. If the expenditure were symmetrically distributed, all data points would lie along the reference line. As shown in the Fig 1, the points in this plot lie above the reference line, demonstrating that the distribution of expenditure is right skewed.

Fig 1. Symmetry plot of the average monthly expenditure


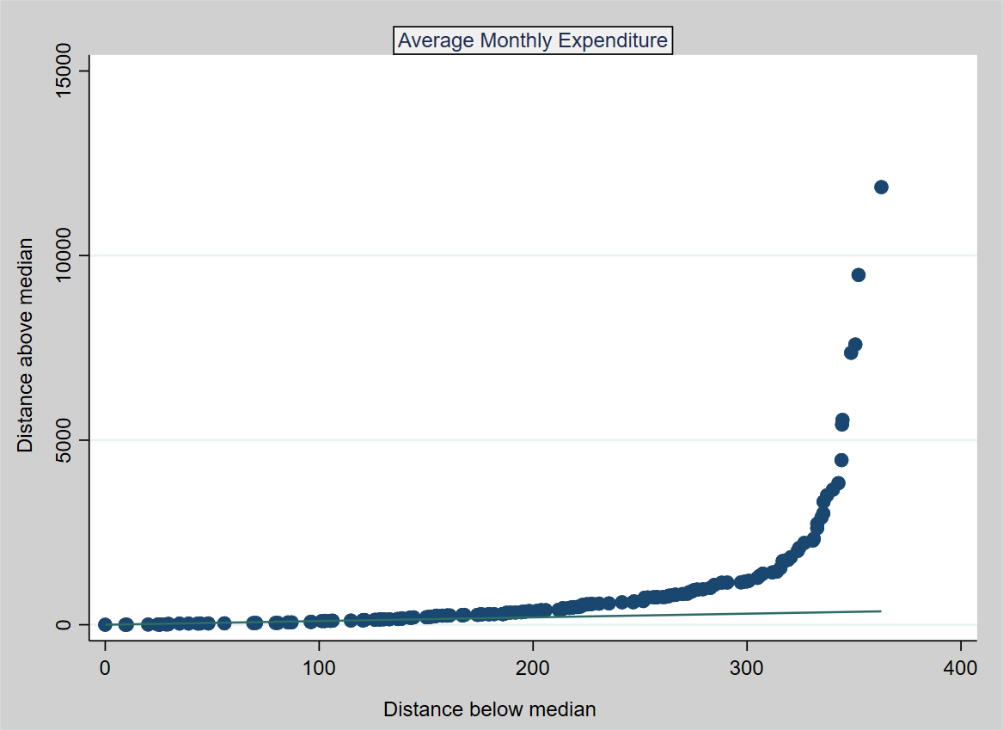


Further to validate this, we used quantile plot below (Fig 2) to ascertain whether the average monthly expenditure were rectangular distributed. If the expenditure were to be rectangular distributed, all the data will be plotted along the line. As shown below, all the points are below the reference line, indicating that the average monthly expenditure distribution is skewed right.

Fig 2. Quantile plot of the average monthly expenditure


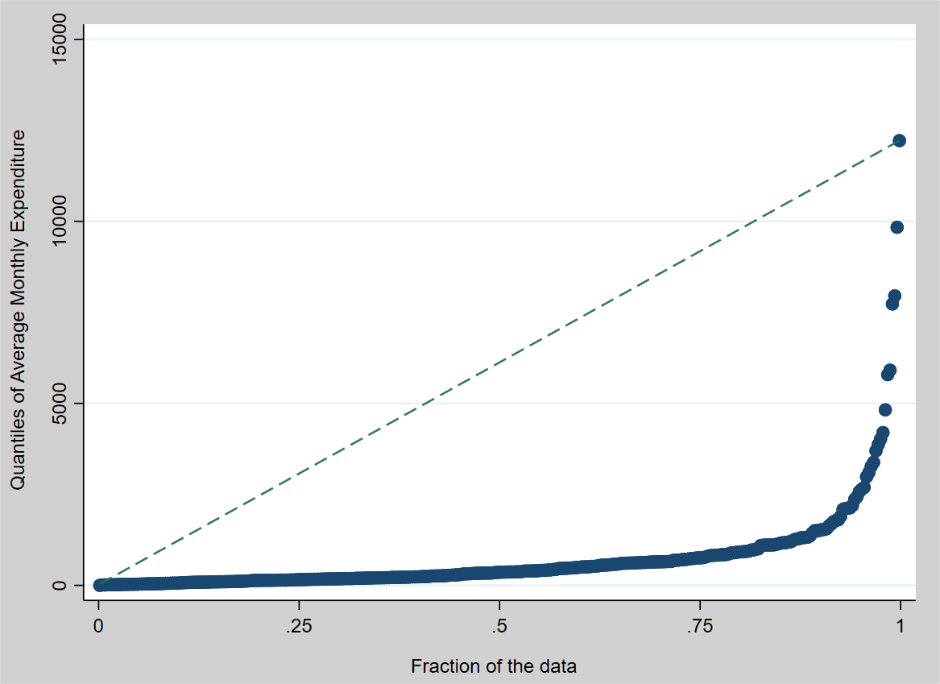


In addition, we performed statistical tests for normality such as the Shapiro-wilk test (W= 0.492, p< 0.0001), Shapiro-Francia test (W’= 0.486, p< 0.0001) and multivariate normality tests with Skewness/Kurtosis test (Pr(Skewness) <0.0001, Pr(Kurtosis)<0.0001), p<0.0001). All the results indicate that we reject the null hypothesis that the average monthly expenditure is normally distributed. Finally, even after using GLM, we ensured to use the model with a smaller AIC = 14.818 (recommended, ([2](#_ENREF_2))) which included the link as log, family as gamma and adjusted for clustering using the pharmacy identity numbers. Thus, these supporting diagnostics validates the choice of GLM.

Reference

1. Mihaylova B, Briggs A, O'Hagan A, Thompson SG. Review of statistical methods for analysing healthcare resources and costs. Health Econ. 2011;20(8):897-916.

2. Akaike H. Information Theory and an Extension of the Maximum Likelihood Principle. In: Parzen E, Tanabe K, Kitagawa G, editors. Selected Papers of Hirotugu Akaike. New York, NY: Springer New York; 1998. p. 199-213.
